# Supplementary material for: Single-cell RNA-seq public data reveal the gene regulatory network landscape of respiratory epithelial and peripheral immune cells in COVID-19 patients
Source: Front Immunol. 2023 Oct 23;14:1194614. doi: 10.3389/fimmu.2023.1194614 (PMC10627007; doi:10.3389/fimmu.2023.1194614)
Supplement: Supplementary file 2 [file Image_1.pdf]

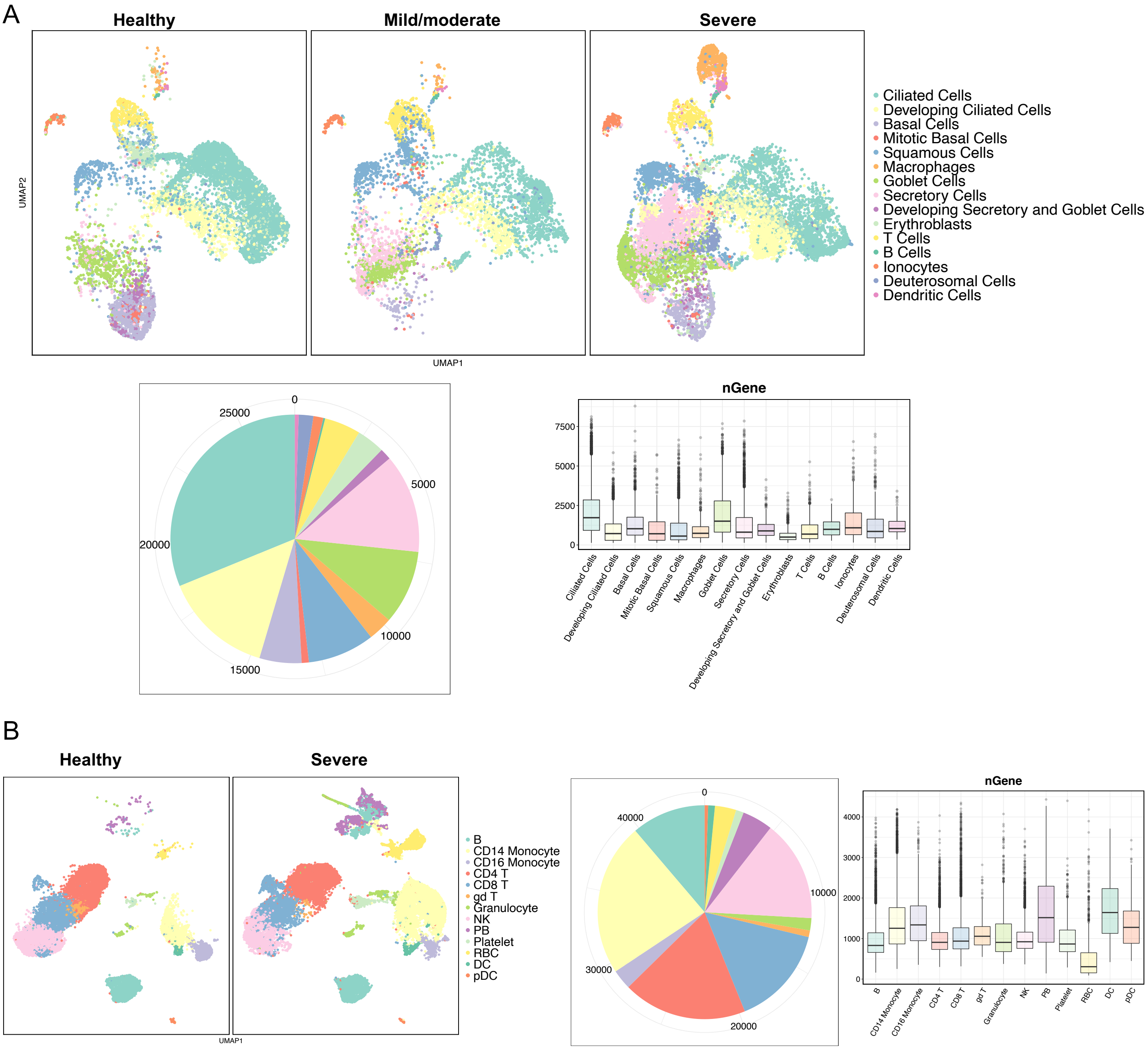

Supplementary Figure S1. Characterization of (A) nasopharyngeal swabs and (B) PBMCs
UMAP plots showing cell types associated with condition (healthy, mild/moderate, and severe). The pie plots indicate the total number of cells used in the study, and the bar plots show the number of genes in each cell type. The lower and upper hinges of the box represent the first and third quartiles (25th and 75th percentiles), the median is marked within the box, and the dots represent outliers. Upper whisker = 75th percentile + 1.5* interquartile range (IQR). Lower whisker = 25th percentile – 1.5*IQR.

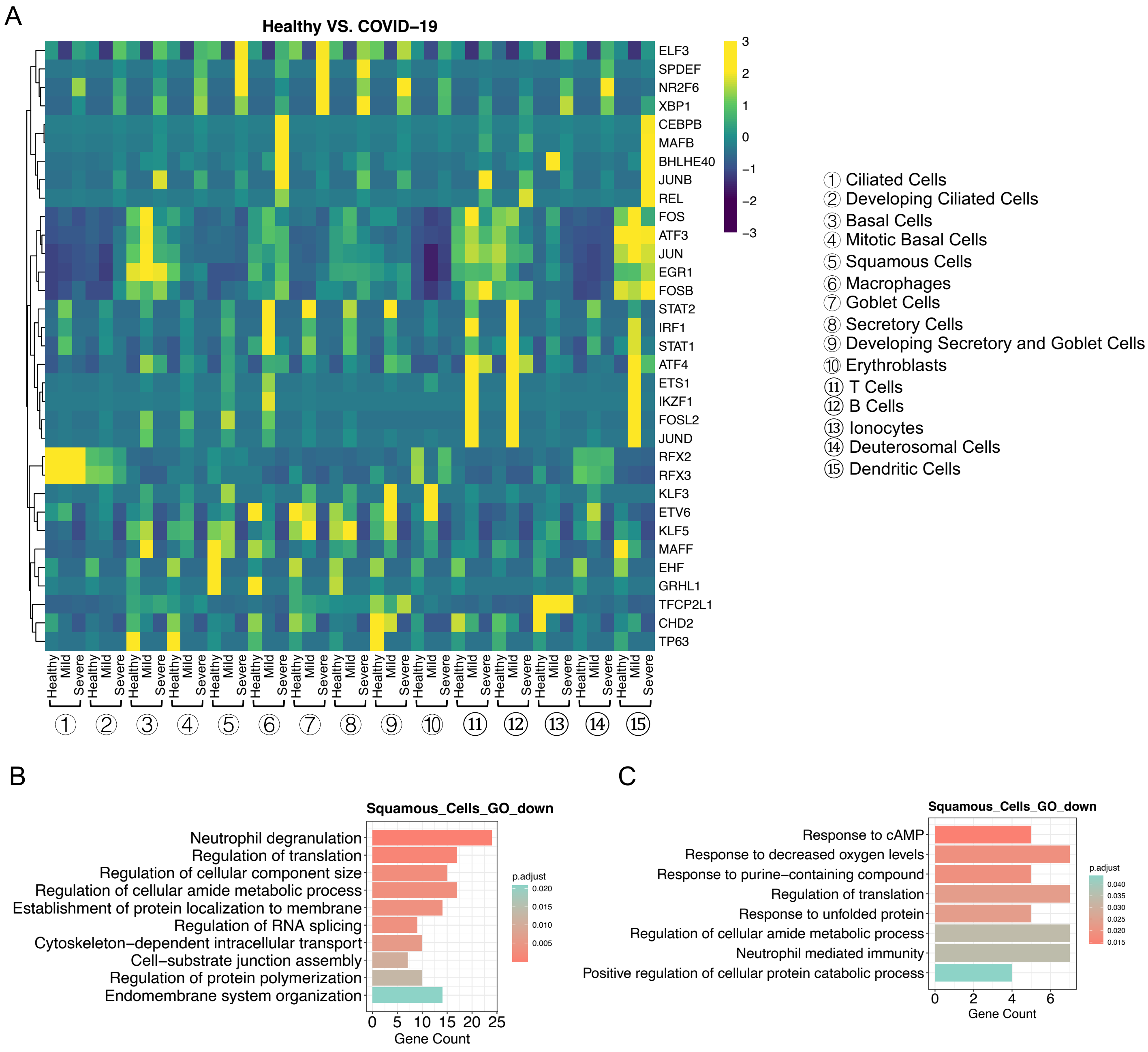

Supplementary Figure S2. Characterization of nasopharyngeal swabs data

(A) Heatmap of area under the curve (AUC) scores of regulons estimated per cell type by SCENIC. Detected regulons are represented by their corresponding transcription factors in the right-hand columns. (B) GO (biological process) enrichment analysis using downregulated genes to compare mild/moderate to healthy cells. (C) GO enrichment analysis using downregulated genes to compare severe to healthy cells.

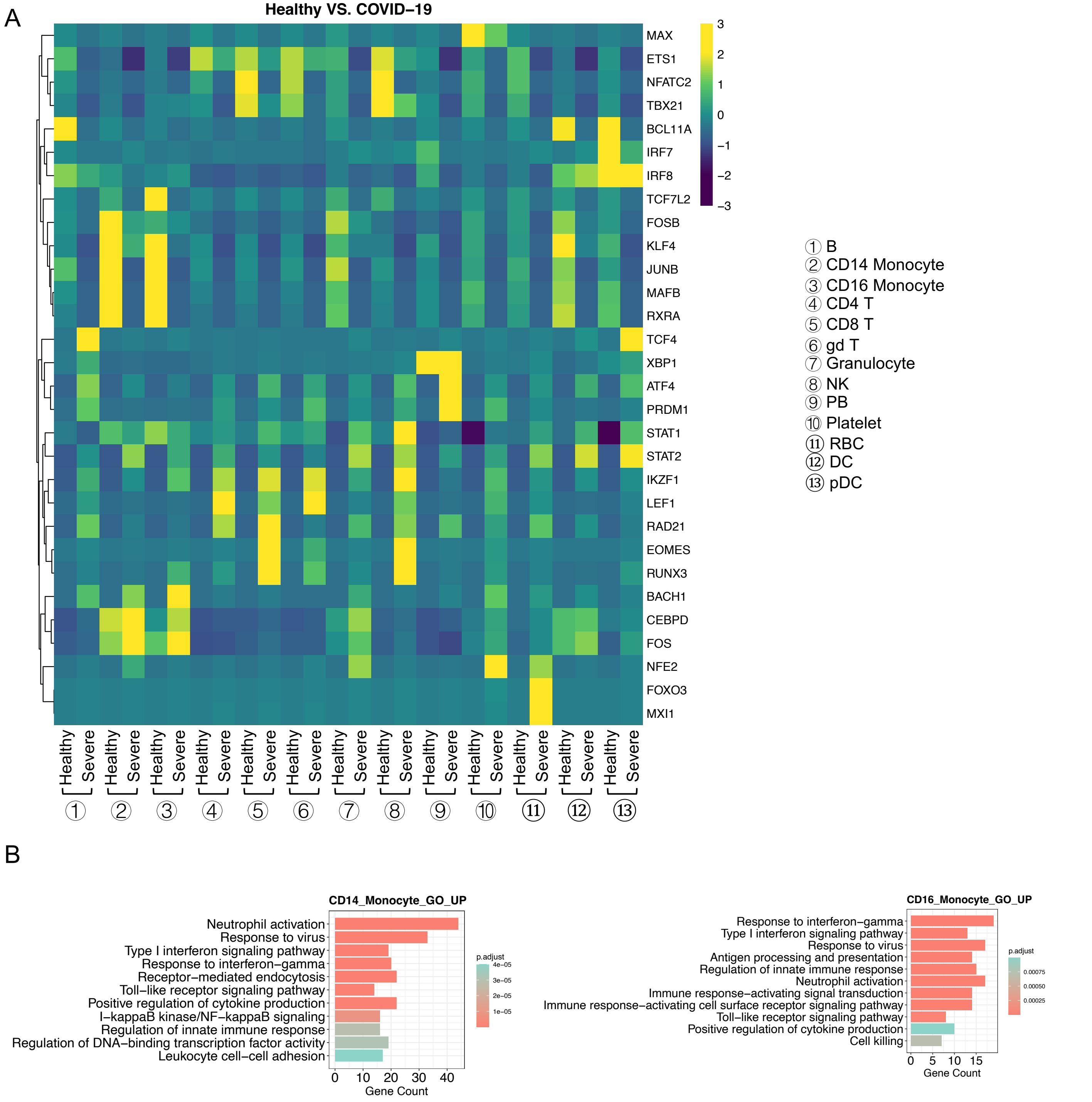

Supplementary Figure S3. Characterization of PBMC data

(A) Heatmap of area under the curve (AUC) scores of regulons estimated per cell type by SCENIC. Detected regulons are represented by their corresponding transcription factors in the right-hand columns.

(B) GO enrichment analysis using downregulated genes to compare severe to healthy cells.

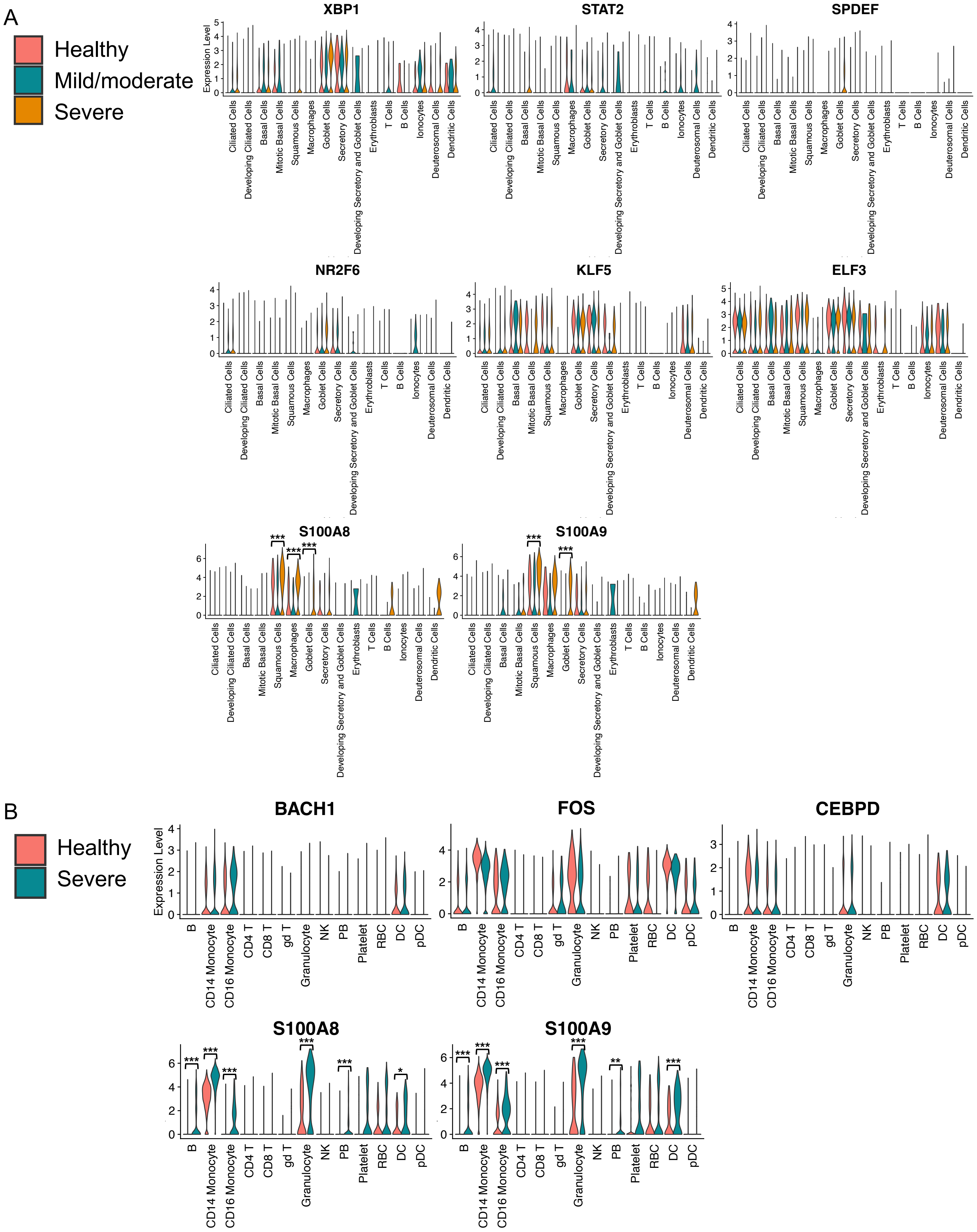

Supplementary Figure S4. Expressions of selected genes for nasopharyngeal swabs (A) and PBMC (B) data

The violin plot shows gene expressions across cell types and conditions (healthy, mild/moderate, and severe COVID-19). Significant expression levels of S100A8/A9 when comparing healthy and severe COVID-19 patients are shown as follows: * $P < 0.05$, ** $P < 0.01$, *** $P < 0.001$.

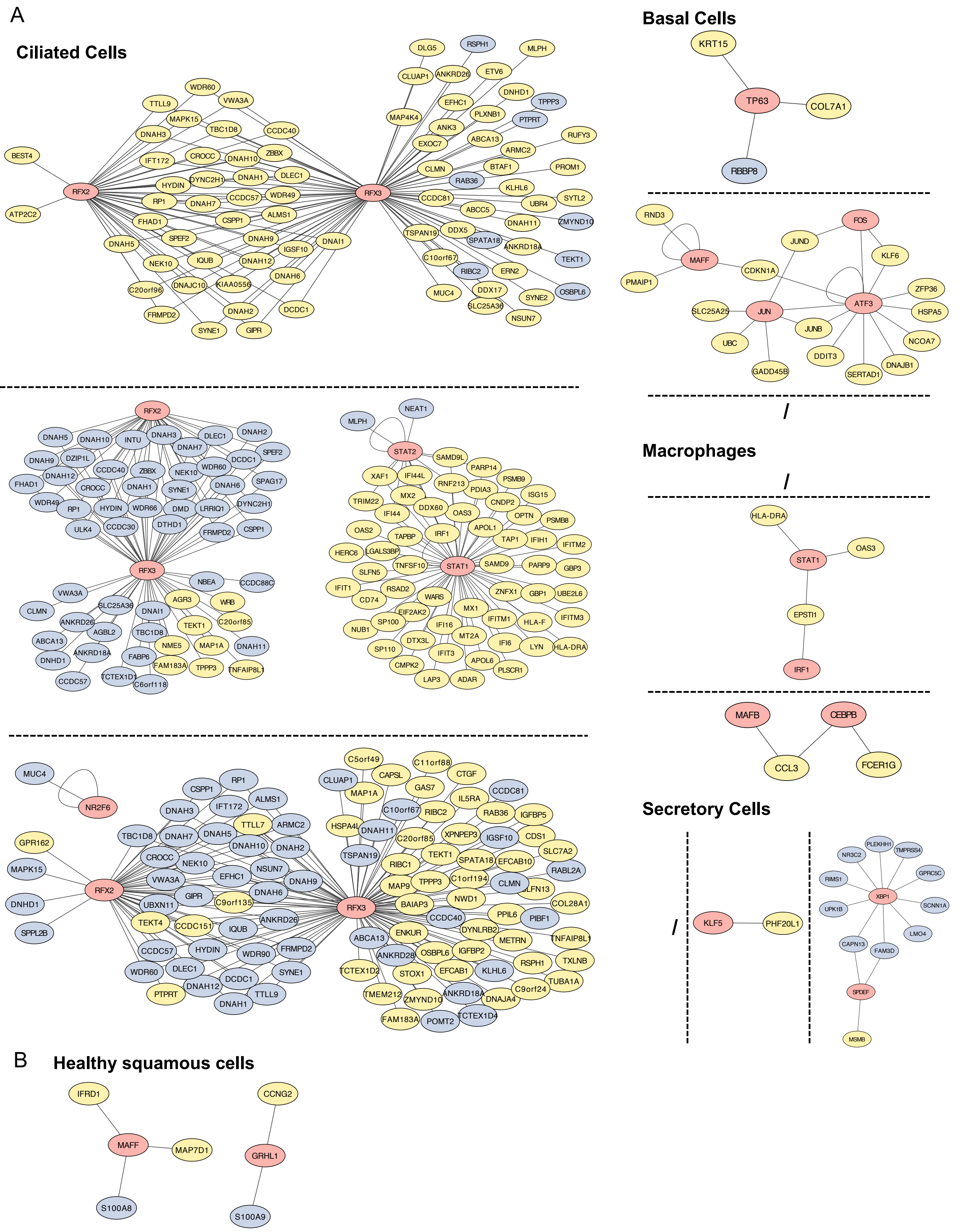

Supplementary Figure S5. Gene regulatory networks of cell types from nasopharyngeal swabs (A) Each panel shows the GRNs in healthy, mild/moderate, and severe COVID-19 from top to bottom, respectively. The slash symbol represents no observed regulons or DEGs. (B). GRNs of healthy squamous cells. In the network, the red, yellow, and light-blue colors represent TF, upregulated, and downregulated genes, respectively.

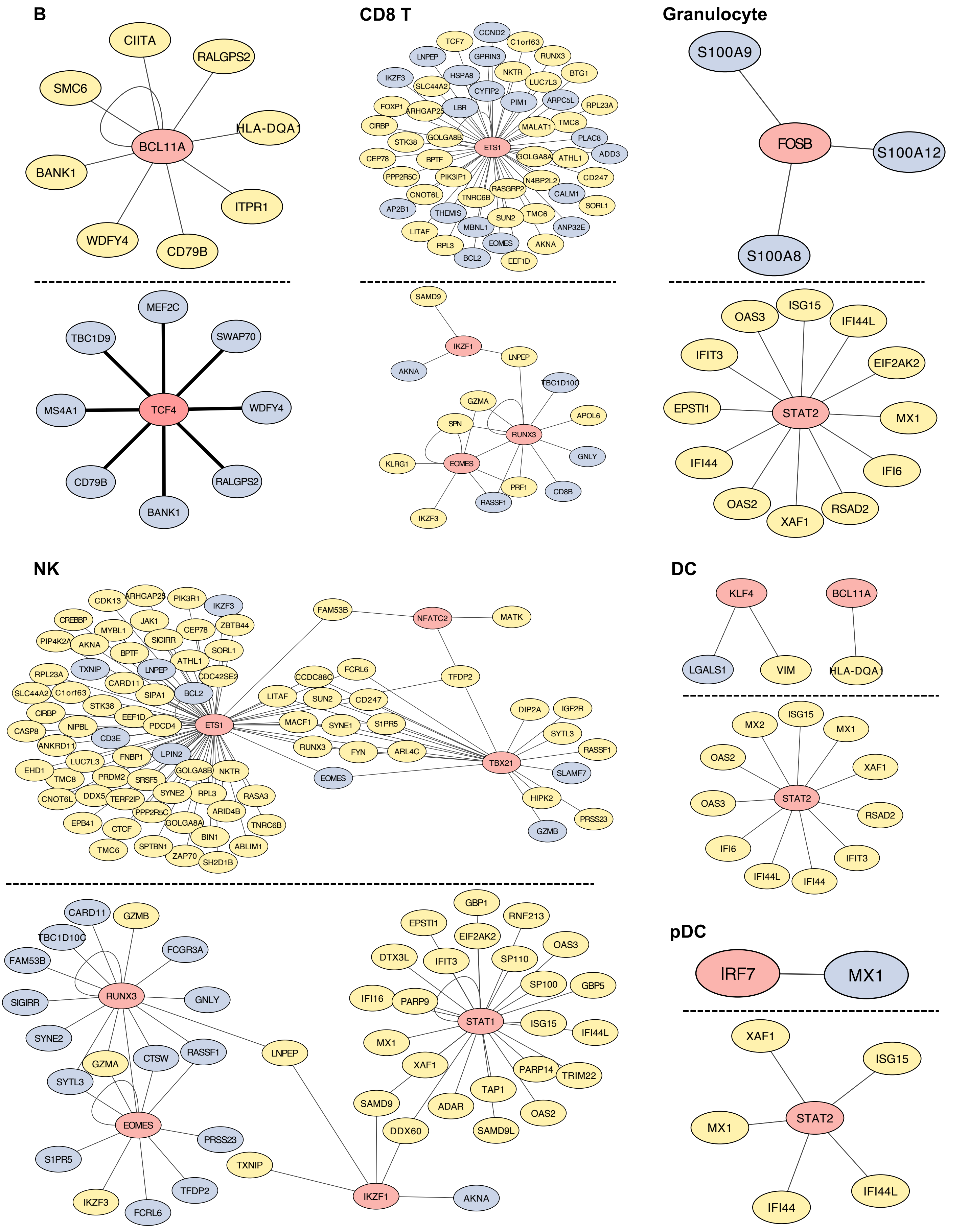

Supplementary Figure S6. Gene regulatory networks of cell types from PBMCs

Each panel represents the GRNs in healthy and severe COVID-19 groups from top to bottom, respectively. The slashed symbol represents no observed regulons or DEGs. In the network, the red, yellow, and light-blue colors represent TF, upregulated, and downregulated genes, respectively.

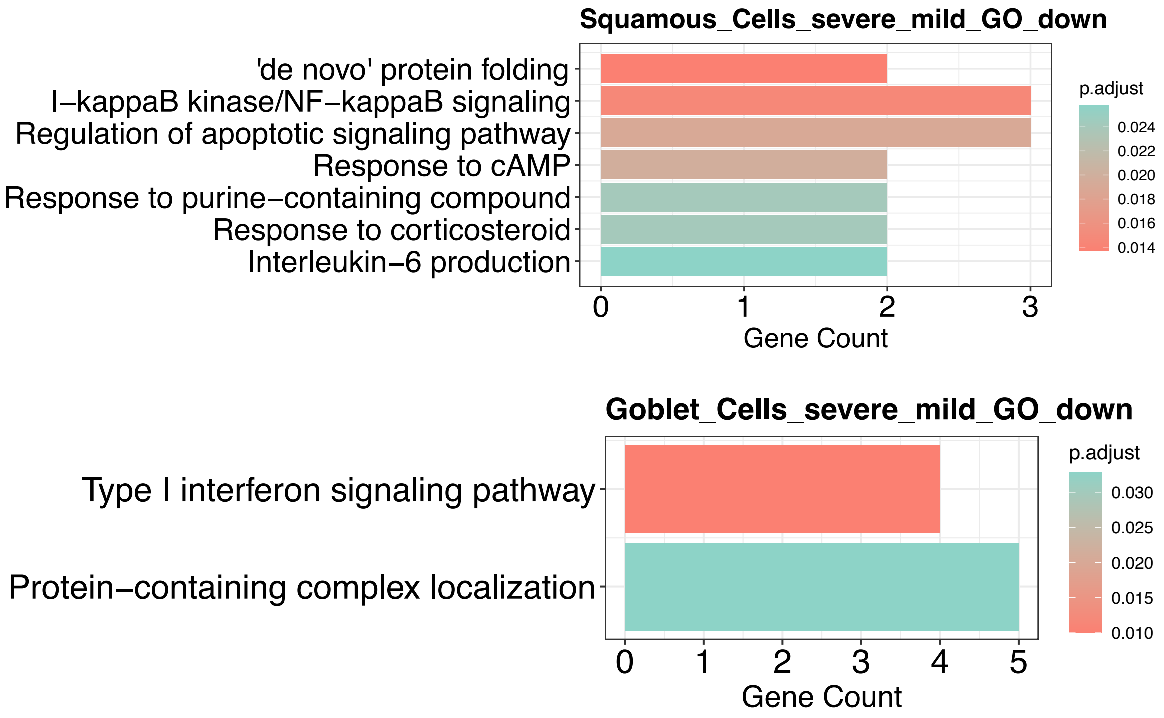

Supplementary Figure S7. GO analysis of downregulated genes in squamous and goblet cells
The GO enrichment analysis is based on the biological processes and identified downregulated genes when comparing severe and mild/moderate COVID-19 patients.

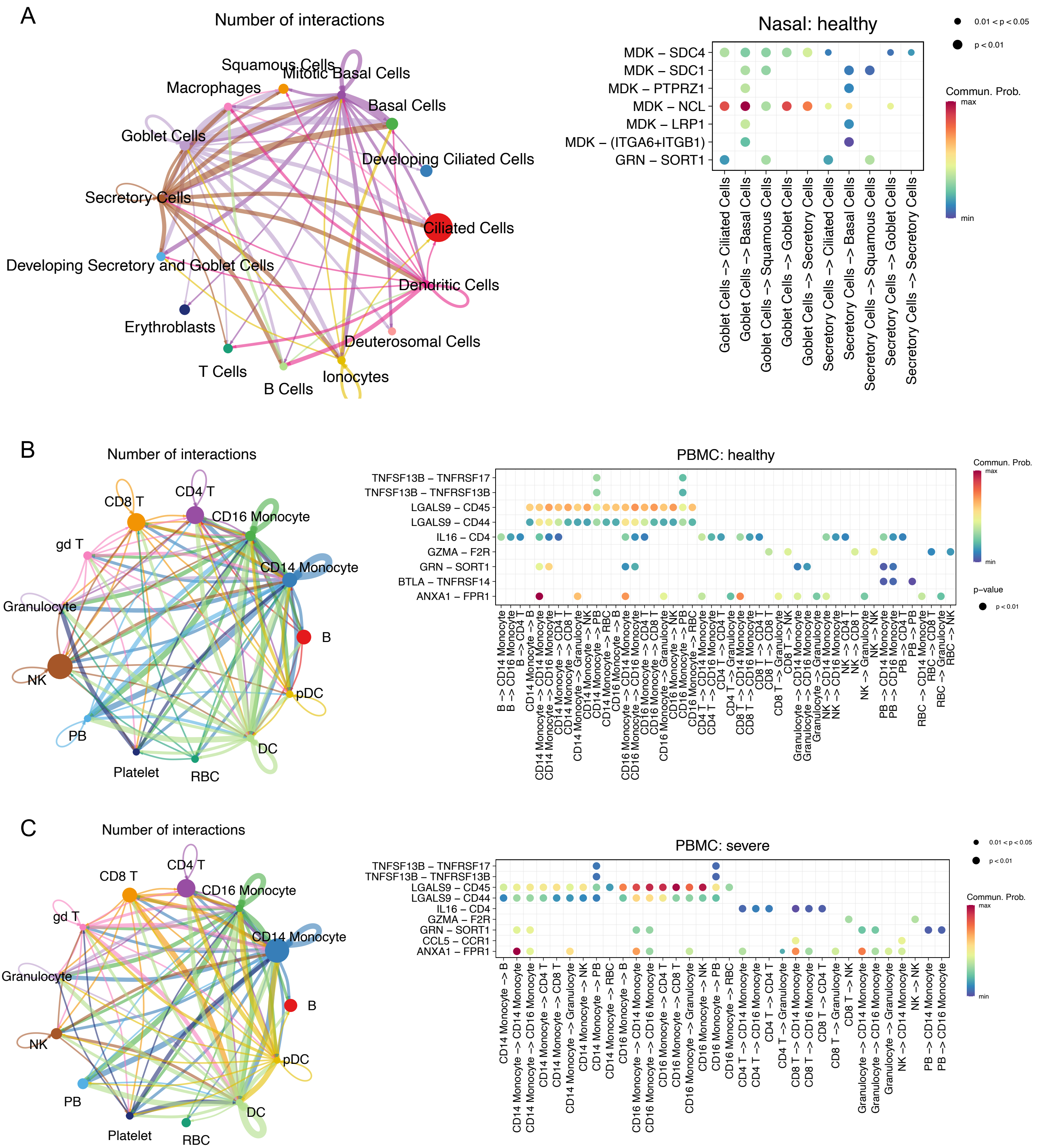

Supplementary Figure S8. CCC analysis using nasopharyngeal swab and PBMC data

(A) CCC analysis of healthy cells from nasopharyngeal swabs. (B) and (C) represent CCC analysis of healthy and severe COVID-19 cells from PBMCs, respectively. Only significant L–R interactions associated with signaling pathways are shown in bubble plots.

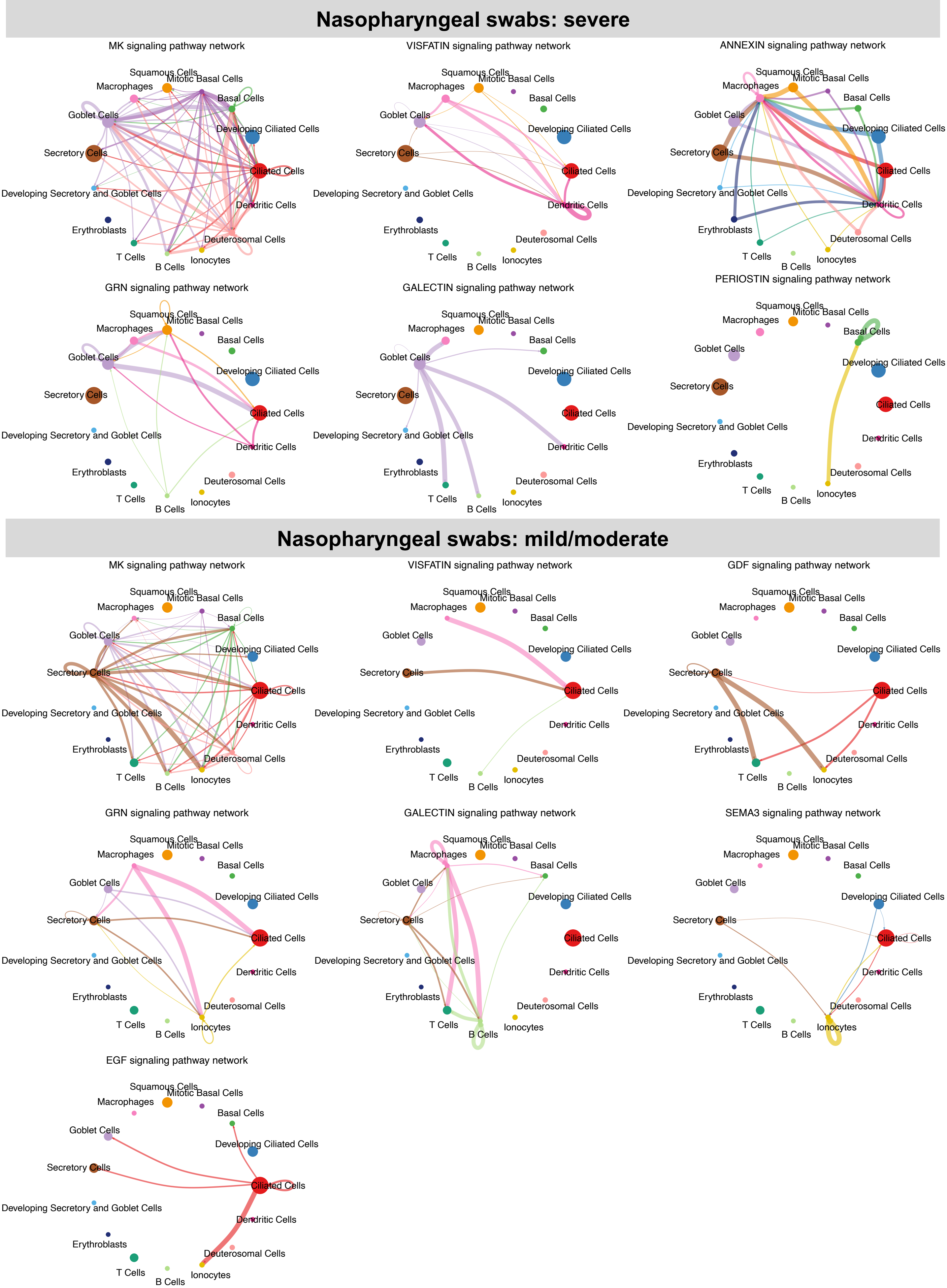

Supplementary Figure S9. Representative signaling pathway networks in nasopharyngeal swabs with severe or mild/moderate COVID-19.

CellChat tool was used to identify significant L–R pairs associated with signaling pathways. Each significantly enriched pathway was visualized in a network.

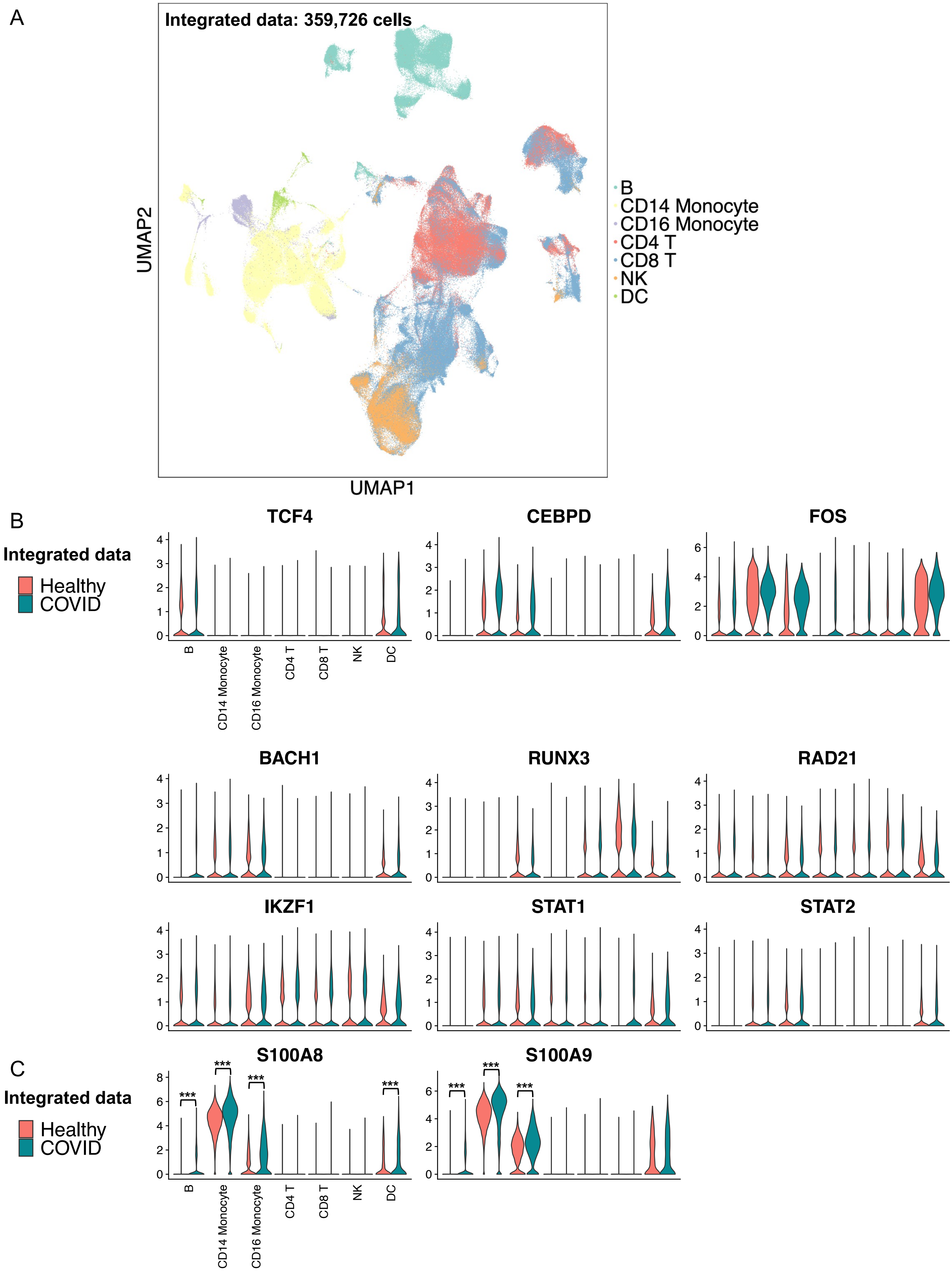

Supplementary Figure S10. Validations by integrating GSE158055 with existing scRNA-seq PBMC data.
(A) UMAP plots showed the integrated immune cells. Immune cells were extracted from GSE158055 and then integrated with PBMCs scRNA-seq data. Common cell types among the datasets were used, including seven immune cells. (B) The violin plots showed relative expressions of identified TFs across cell types under severe condition. (C) The violin plots represented differentially expressed S100A8/A9 across cell types. Significance: * $P < 0.05$, ** $P < 0.01$, *** $P < 0.001$.

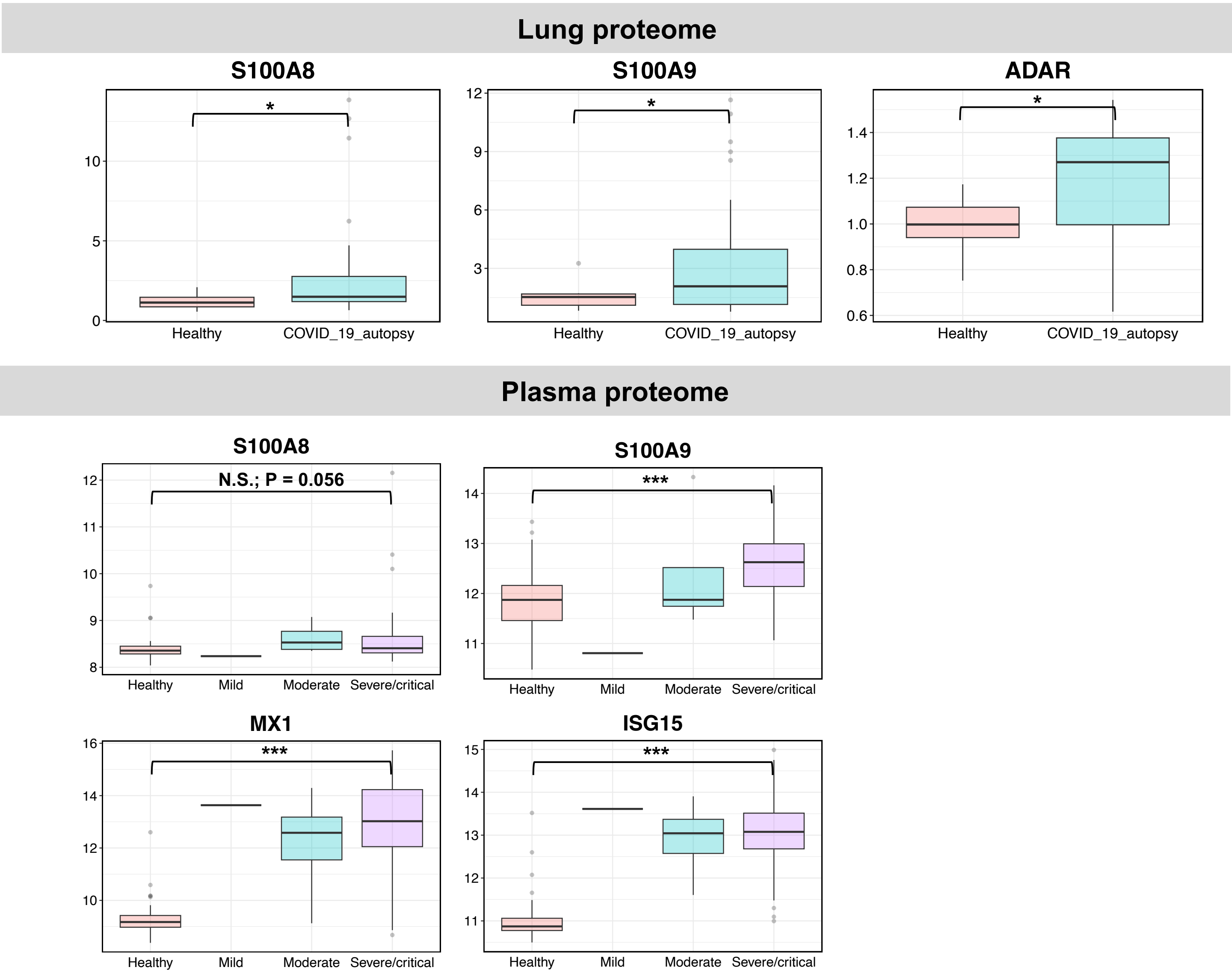

Supplementary Figure S11. Significantly abundant proteins of cytokines/ISGs/S100 family in COVID-19 cases using lung and plasma proteomes.

The box plots represented protein levels in lung and plasma proteomes. The upregulated cytokines, ISGs, and S100A8/A9 in autopsy or severe/critical COVID-19 cases, as presented in Table S13, were utilized to validate their corresponding protein abundances after SARS-CoV-2 infection. A Student t-test was conducted to identify significant differences when comparing COVID-19 cases (either autopsy or severe/critical) to healthy donors. A cutoff of $P < 0.05$ was used to determine significance. Significance: * $P < 0.05$, ** $P < 0.01$, *** $P < 0.001$.

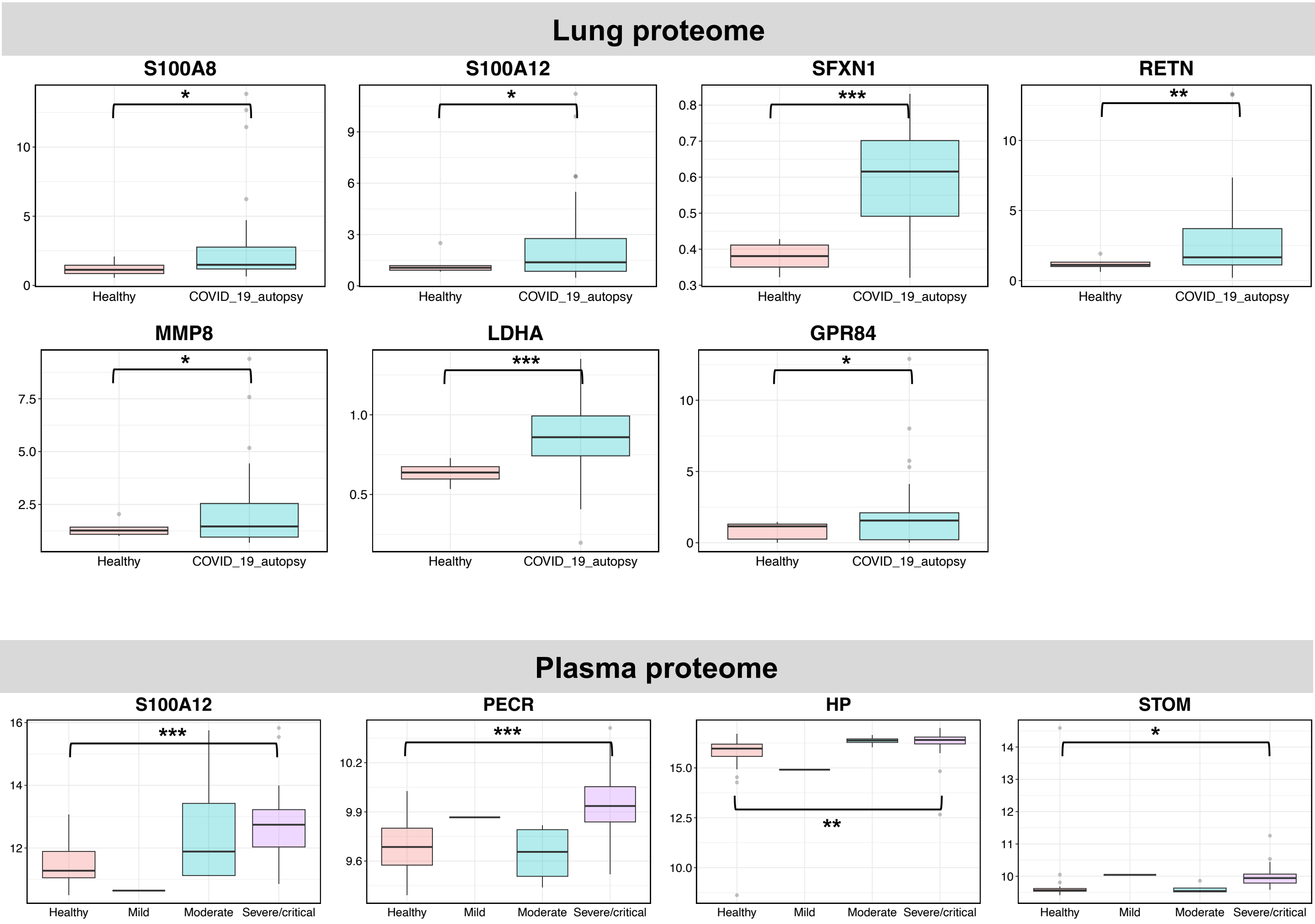

Supplementary Figure S12. Significantly abundant proteins among the top 26 genes in COVID-19 cases using lung and plasma proteomes.

The top 26 genes identified in Figure 5 were utilized to validate their corresponding protein abundances after SARS-CoV-2 infection. A Student t-test was conducted to identify significant differences when comparing the protein levels between autopsy or severe/critical COVID-19 cases and healthy donors. A cutoff of $P < 0.05$ was used to determine significance. Significance: * $P < 0.05$, ** $P < 0.01$, *** $P < 0.001$.
